# Integrative Natural Approaches for Age-Related Testosterone Decline: A Synergistic Framework Combining Exercise, Nutrition, and Bioactive Compounds

**DOI:** 10.7759/cureus.101276

**Published:** 2026-01-11

**Authors:** Inna Shypilova, Olena Bolgova, Volodymyr Mavrych

**Affiliations:** 1 Department of Medical Biochemistry, St. Matthew's University, West Bay, CYM; 2 College of Medicine, Alfaisal University, Riyadh, SAU

**Keywords:** diet, integrative interventions, leydig cells, oxidative stress, resistance training, supplements, testosterone decline

## Abstract

Age-related testosterone decline significantly affects metabolism, cardiovascular health, bone density, muscle mass, and psychological well-being in aging men. While testosterone replacement therapy offers direct supplementation, safety concerns have prompted growing interest in natural approaches to preserving endogenous production.

The underlying pathophysiology involves disruptions of the hypothalamic-pituitary-gonadal axis, affecting both central and peripheral mechanisms. At the testicular level, microenvironmental alterations include Leydig cell decline, oxidative stress, inflammatory upregulation, and mitochondrial dysfunction, all of which contribute to reduced steroidogenic capacity.

Addressing these mechanisms requires multifaceted interventions, beginning with targeted exercise strategies. Resistance training proves most effective when employing progressive overload and compound exercises, while combined resistance-aerobic protocols offer synergistic benefits that exceed single-modality training by simultaneously targeting cardiovascular health and muscle preservation.

Complementing these exercise interventions, Mediterranean dietary patterns emphasizing whole foods, healthy fats, and antioxidant-rich plants support endogenous testosterone production while reducing systemic inflammation. Furthermore, micronutrient optimization, particularly zinc and vitamin D, proves essential for maintaining steroidogenic enzyme function.

Beyond conventional nutrition, emerging evidence supports the use of plant-derived bioactive compounds for hormonal optimization. Antioxidants such as resveratrol, curcumin, and quercetin reduce oxidative stress and protect steroidogenic enzymes, while adaptogenic herbs, particularly ashwagandha, support testosterone levels by modulating cortisol and promoting testicular health.

Collectively, integrative approaches that combine strategic exercise programming, nutrient-dense dietary patterns, and targeted bioactive compounds show considerable promise in addressing age-related declines in testosterone through complementary mechanisms. These strategies offer favorable safety profiles compared with pharmaceutical interventions, providing sustainable pathways for healthy aging and hormonal optimization in the aging male population.

## Introduction and background

Contemporary societies exhibit delayed reproductive age and increased life expectancy, creating unprecedented challenges for male endocrine health throughout extended lifespans [1]. While the male reproductive system exhibits relatively delayed aging compared with the female reproductive system, increasing age substantially impairs its function, with age-related testosterone decline representing a characteristic manifestation of this process [1,2]. Testosterone, a crucial male sex hormone, plays pivotal roles not only in spermatogenesis and sexual function but also in metabolism, psychology, and cardiovascular health [2,3]. The aging process exerts profound effects on the hypothalamic-pituitary-gonadal axis and Leydig cells, leading to reduced testosterone levels that adversely affect male health across multiple domains [1,4].

The biological foundations of age-related testosterone decline reflect the complex interplay between evolutionary adaptation and physiological deterioration [5]. Reproductive endocrine function is inherently subject to change as men age, with hormonal variations resulting from general somatic decline [5]. However, these changes demonstrate considerable individual variability and can differ significantly between human male populations depending on lifestyle and ecological context [5,6]. Recent evolutionary anthropology research has provided valuable insights into these adaptive mechanisms, though the degree to which testosterone variation represents adaptive responses remains an active area of investigation [5].

Understanding the origins and comparative context of reproductive endocrine function in older human men offers significant benefits for the development of targeted interventions [1,7]. While exogenous testosterone supplementation can partially ameliorate age-related testosterone deficiency, concerns about long-term safety have prompted increased interest in preserving endogenous testosterone production capacity during aging [1,2]. This review synthesizes current evidence on integrative natural approaches combining exercise interventions, nutritional strategies, and bioactive compounds to address age-related testosterone decline through synergistic mechanisms [7,8].

## Review

Methods

This narrative review synthesized evidence on integrative natural approaches for age-related testosterone decline. We conducted searches in PubMed, Scopus, and Web of Science (2015-2025) using the following search terms: ("testosterone" OR "androgen") AND ("aging" OR "age-related decline") AND ("exercise" OR "resistance training" OR "diet" OR "nutrition" OR "Mediterranean diet" OR "supplements" OR "phytochemicals" OR "natural interventions"). Additional references were identified through citation tracking of key articles. We prioritized recent systematic reviews, randomized controlled trials, and observational studies in human populations. Studies involving testosterone replacement therapy as the primary intervention were excluded. This narrative synthesis aimed to provide a comprehensive overview of evidence-based natural approaches to maintain the proper testosterone level in elderly men.

Mechanisms of age-related testosterone decline

Hypothalamic-Pituitary-Gonadal Axis Disruptions

Age-related testosterone decline involves complex disruptions throughout the hypothalamic-pituitary-gonadal axis that extend beyond simple testicular dysfunction [4]. Golan et al. elucidated that age-related hypogonadism reflects a combination of primary testicular failure and secondary hypothalamic-pituitary axis dysfunction, highlighting the multifactorial mechanisms underlying acquired androgen deficiency in aging men [4]. Their findings revealed that aging affects multiple levels of hormonal regulation, with both central and peripheral mechanisms contributing to the decline in testosterone [4,6].

The hypothalamic-pituitary component of testosterone regulation is particularly vulnerable to aging-related changes [1,2]. Gonadotropin-releasing hormone secretion patterns become altered with advancing age, leading to modified luteinizing hormone plasticity and reduced responsiveness to testosterone feedback mechanisms [1]. These central changes interact with peripheral alterations in testicular function, creating a complex pathophysiology that requires multifaceted intervention approaches [6,9].

Longitudinal studies have provided crucial insights into the temporal patterns of testosterone decline [10]. Fabbri et al. reported that bioavailable testosterone declines linearly across a wide age range in men and women from the Baltimore Longitudinal Study of Aging [10]. Their analysis demonstrated that the magnitude of longitudinal decline in testosterone levels from baseline shows individual variability, influenced by factors including genetic polymorphisms, lifestyle variables, and overall health status [6,10,11].

Testicular Microenvironment and Leydig Cell Function

The testicular microenvironment undergoes significant alterations with aging that directly impact testosterone biosynthesis [1]. Leydig cells, the primary source of testosterone synthesis in men, experience both numerical reduction and functional decline with aging [1]. These changes result from multiple interconnected mechanisms, including oxidative stress accumulation, upregulation of inflammatory mediators, and mitochondrial dysfunction [1,12].

Aging affects testosterone synthesis through various pathways, including alterations in enzyme activities involved in steroidogenesis, reduced substrate availability, and impaired cellular energy metabolism [1]. The testicular microenvironment also undergoes changes in vascular supply, interstitial inflammation, and local growth factor availability, all of which contribute to diminished testosterone production capacity [1]. Understanding these mechanisms provides a foundation for targeted interventions aimed at preserving or restoring endogenous testosterone production [1,7].

Cheng et al. provided a comprehensive analysis of age-related mechanisms of testosterone decline and intervention strategies, emphasizing that aging exerts profound effects on the hypothalamic-pituitary-gonadal axis and Leydig cells, precipitating testosterone decline that adversely affects male health [1]. Their review highlighted the importance of addressing multiple pathways simultaneously through integrative approaches rather than focusing on single intervention modalities [1,8].

Psychosocial Stress and Hormonal Dysregulation

Psychological and social factors represent critical but often underappreciated contributors to age-related testosterone decline. The bidirectional relationship between psychosocial stress and testosterone creates a self-reinforcing cycle that accelerates hormonal deterioration and negatively impacts overall health in aging men [13]. Chronic psychological stress exerts profound suppressive effects on testosterone production through multiple interconnected mechanisms. When individuals experience prolonged stress, the hypothalamic-pituitary-adrenal axis becomes chronically activated, leading to sustained elevation of cortisol levels [14]. Cortisol and testosterone demonstrate an inverse relationship, with elevated cortisol directly inhibiting testosterone synthesis through several pathways [15]. Social isolation and loneliness represent increasingly prevalent chronic stressors with direct neuroendocrine consequences. Epidemiological studies demonstrate that socially isolated men exhibit significantly altered hormonal profiles compared with socially connected individuals [16,17]. While social isolation can reduce testosterone levels through chronic stress activation, low testosterone itself may reduce social motivation and engagement, creating a vicious cycle of withdrawal and further hormonal decline [18,19]. Men with multiple sources of emotional social support demonstrate healthier testosterone profiles, suggesting that social connectivity may serve as a protective factor against age-related decline [20]. The relationship between testosterone and mood disorders is bidirectional and clinically significant. Epidemiological studies consistently demonstrate that men with depression exhibit lower circulating testosterone levels compared to nondepressed cohorts [21]. The basolateral amygdala and medial prefrontal cortex play a significant role in anxiety disorders and chronic fear conditions [22]. Testosterone influences mood regulation through multiple neurotransmitter systems, including serotonin, dopamine, and norepinephrine pathways [23,24]. Testosterone generally exhibits an anxiolytic (anxiety-reducing) effect by modulating stress response systems, but this can vary based on individual factors, the nature of the stress, and the developmental stage [25].

Exercise interventions for testosterone optimization

Resistance Training Protocols

Resistance training represents one of the most extensively studied and effective natural interventions for maintaining testosterone levels in aging men [8,26,27]. The acute and chronic effects of resistance exercise on testosterone production involve complex physiological mechanisms that extend beyond simple hormonal stimulation [28]. Resistance training protocols typically demonstrate optimal effectiveness when incorporating progressive overload principles, adequate recovery periods, and appropriate training volumes [8,27,28].

Contemporary research indicates that the effectiveness of resistance training for testosterone optimization depends heavily on program design variables, including exercise selection, training intensity, volume distribution, and recovery protocols [8,28]. Multijoint compound exercises appear particularly effective at stimulating testosterone release compared with single-joint movements, likely due to greater recruitment of overall muscle mass and greater metabolic demand [8,28]. Training intensities ranging from moderate to high show beneficial effects, though the optimal intensity prescription may vary based on individual characteristics and baseline fitness levels [8,27].

The temporal patterns of testosterone response to resistance training reveal both immediate postexercise increases and longer term adaptations that develop over weeks to months of consistent training [8,27,29]. These adaptations involve improvements in Leydig cell responsiveness, enhanced luteinizing hormone sensitivity, and optimization of the hypothalamic-pituitary-gonadal axis function [1,8]. Understanding these temporal patterns helps inform optimal program progression and periodization strategies for sustained testosterone optimization [8,28].

Aerobic Exercise Considerations

Aerobic exercise interventions for testosterone optimization require careful consideration of intensity, duration, and frequency parameters to maximize benefits while avoiding potential negative effects [8,13,17]. Moderate-intensity aerobic exercise generally produces favorable effects on testosterone levels and overall hormonal balance, while excessive endurance training may lead to suppressed testosterone production through multiple mechanisms [8,30].

The relationship between aerobic exercise and testosterone demonstrates a complex dose-response pattern that varies considerably between individuals [8,26]. Factors such as baseline fitness level, training history, nutritional status, and recovery capacity influence the testosterone response to aerobic exercise interventions [8,30]. Optimal protocols typically emphasize moderate intensities performed for reasonable durations with adequate recovery between sessions [8,26].

Physical activity interventions have demonstrated effectiveness in addressing age-related changes in sedentary men [7, 26]. Abdel-Sater examined testosterone levels in long-term sedentary aging men and the effects of antiaging strategies, finding that structured physical activity programs could produce meaningful improvements in hormonal profiles when implemented with appropriate progression and consistency [7].

Combined Training Approaches

Integrating resistance and aerobic training modalities offers potential synergistic benefits for testosterone optimization that exceed the effects of either modality alone [8,30]. Combined training approaches allow for targeting multiple physiological systems simultaneously while providing variety that may enhance long-term adherence to exercise interventions [8,31].

The optimal integration of resistance and aerobic training requires careful consideration of interference effects, recovery requirements, and individual capacity limitations [8,30]. Concurrent training protocols that emphasize appropriate sequencing, intensity distribution, and recovery management can produce favorable hormonal adaptations while improving overall health and functional capacity [8,31].

Research suggests that combined training approaches may be particularly beneficial for addressing the multifaceted nature of age-related testosterone decline by simultaneously targeting cardiovascular health, muscle mass preservation, metabolic function, and hormonal optimization [8,32]. These comprehensive benefits support the implementation of combined training protocols as foundational components of integrative natural approaches to testosterone optimization [7,8].

Nutritional strategies and dietary patterns

Macronutrient Considerations

Optimal macronutrient intake is crucial for supporting endogenous testosterone production through multiple physiological pathways [33-35]. Adequate protein intake supports amino acid requirements for steroidogenesis while providing the building blocks needed to maintain lean muscle mass, which correlates positively with testosterone levels [34,36]. Research indicates that protein requirements for older men engaged in exercise programs may exceed standard recommendations to optimize both muscle protein synthesis and hormonal function [34,35].

Carbohydrate intake influences testosterone production by modulating insulin signaling, cortisol regulation, and overall energy availability for steroidogenic processes [33,34]. Moderate carbohydrate intake appears optimal for testosterone production, as both excessive restriction and excessive consumption may negatively affect hormonal balance [34]. The timing of carbohydrate intake relative to exercise sessions may also influence testosterone responses by altering cortisol dynamics and recovery processes [8,33].

Dietary fat intake shows particularly strong relationships with testosterone production, as cholesterol is the primary substrate for steroidogenesis [34]. However, the type and quality of dietary fats significantly influence these relationships, with emphasis on omega-3 fatty acids, monounsaturated fats, and adequate saturated fat intake supporting optimal testosterone production while promoting overall cardiovascular health [33,36,37].

Mediterranean Diet Patterns

Mediterranean dietary patterns have received increasing attention for their potential benefits in supporting male reproductive health and hormonal function [34-41]. These dietary approaches emphasize whole foods, adequate intake of healthy fats, antioxidant-rich plant foods, and moderate portions of high-quality protein sources, creating nutritional environments that support endogenous testosterone production [39,40].

The anti-inflammatory properties of Mediterranean dietary patterns may contribute to their beneficial effects on testosterone levels by reducing systemic inflammation that can interfere with steroidogenesis [38,39]. Additionally, the high antioxidant content of these dietary patterns helps combat oxidative stress, which represents a significant contributor to age-related testosterone decline and testicular dysfunction [34,38].

Research examining Mediterranean dietary patterns and male reproductive health has demonstrated positive associations with sperm parameters, hormonal profiles, and overall reproductive function [39-41]. These findings support the implementation of Mediterranean-style dietary approaches as components of comprehensive natural interventions for age-related testosterone decline [31,39,42]. While traditional Mediterranean patterns provide adequate protein for general health, optimization for aging men may require targeting higher protein intakes (1.2-1.6 g/kg/day) distributed across meals to overcome age-related anabolic resistance and support muscle protein synthesis, particularly when combined with resistance training interventions [35].

Micronutrient Optimization

Specific micronutrients play essential roles in testosterone biosynthesis and overall reproductive health, making targeted optimization of these nutrients a crucial component of natural intervention strategies [33,43,44]. Zinc represents perhaps the most well-documented micronutrient for testosterone support, serving as a cofactor in multiple steroidogenic enzymes and demonstrating clear deficiency-related impacts on testosterone production [37,43]. While Mediterranean dietary patterns provide numerous nutritional benefits, they may not consistently deliver optimal zinc levels for testosterone support, particularly in older men with increased requirements, making targeted supplementation a practical consideration for achieving therapeutic doses [43].

Vitamin D status shows strong correlations with testosterone levels across multiple populations, with deficiency states associated with reduced testosterone production and supplementation studies, demonstrating beneficial effects in deficient individuals [33,44]. Given limited dietary sources and variable sun exposure, particularly in older adults, vitamin D supplementation represents a foundational component of most testosterone optimization protocols [44]. The mechanisms underlying vitamin D's effects on testosterone involve both direct actions on testicular tissue and indirect effects through calcium homeostasis and overall health optimization [44].

Magnesium, selenium, and various B vitamins also contribute to optimal testosterone production through their roles in enzyme function, antioxidant protection, and overall metabolic health [33,45]. A comprehensive approach to micronutrient optimization involves both dietary strategies to enhance nutrient density and targeted supplementation when indicated by assessment of individual nutritional status [45,46].

Bioactive Compounds and Phytochemicals

Antioxidant compounds: Oxidative stress represents a primary mechanism underlying age-related testosterone decline, making antioxidant compounds valuable components of natural intervention strategies [1,34,38]. Numerous plant-derived antioxidants have demonstrated potential to support testosterone production by reducing oxidative damage to testicular tissue and protecting steroidogenic enzyme function [34,37].

Resveratrol, a polyphenolic compound found in grape skins and other plant sources, has shown promising effects in preclinical studies for protecting against age-related testosterone decline [47-49]. The mechanisms of resveratrol's effects involve both direct antioxidant actions and activation of cellular longevity pathways that may help preserve testicular function during aging [47,48]. Research examining resveratrol's effects on male reproductive health has shown promising results, though human clinical trials remain limited and warrant further investigation [48,49].

Curcumin, the active compound in turmeric, demonstrates potent anti-inflammatory and antioxidant properties that may support testosterone production by reducing systemic inflammation and oxidative stress [47]. Research examining curcumin's effects on male reproductive health has shown promising results, though human clinical trials remain limited and warrant further investigation [47]. Other antioxidant compounds, including quercetin and various polyphenols, offer additional potential for natural testosterone support [47,50].

Adaptogenic herbs: Traditional adaptogenic herbs offer potential natural approaches to supporting testosterone levels by modulating stress responses and supporting overall endocrine function [51,52]. These plant compounds typically demonstrate complex mechanisms of action that involve multiple physiological pathways rather than direct hormonal stimulation [51,52].

Ashwagandha has received particular attention for its potential testosterone-supporting effects, with several clinical studies demonstrating beneficial effects on testosterone levels and related parameters, such as muscle strength and stress markers [51-53]. The mechanisms of ashwagandha's effects appear to involve modulation of cortisol levels and direct support of testicular function [51].

Other adaptogenic compounds, including *Eurycoma longifolia*, Rhodiola, and *Tribulus terrestris*, have shown varying degrees of promise in preliminary research for supporting male hormonal health [51,52]. However, the quality and consistency of research on these compounds vary considerably, necessitating careful evaluation of evidence quality when considering their implementation [51].

Quercetin and flavonoids: Flavonoid compounds, particularly quercetin, have demonstrated potential to support testosterone production through multiple mechanisms, including aromatase inhibition, antioxidant protection, and anti-inflammatory effects [34,37,47]. These actions may help preserve testosterone levels by reducing conversion to estrogen while protecting testicular tissue from age-related damage [37,47].

Quercetin's effects on testosterone involve both direct and indirect mechanisms, including protection of Leydig cells from oxidative damage and potential inhibition of enzymes involved in testosterone metabolism [50]. Research examining quercetin supplementation in aging men has shown promising preliminary results, though larger clinical trials are needed to establish optimal dosing protocols and confirm efficacy [50].

The broader category of flavonoid compounds offers numerous potential candidates for natural testosterone support, with different compounds demonstrating varying mechanisms of action and potency [37,47]. A comprehensive approach to flavonoid intake, including both dietary sources and targeted supplementation, may provide synergistic benefits for testosterone optimization [41,47].

Discussion

Mechanistic Synergies

The combination of exercise interventions, nutritional optimization, and bioactive compound supplementation creates opportunities for synergistic effects that exceed the benefits of individual interventions [8,35,45]. Exercise enhances nutrient utilization and may improve the bioavailability of certain compounds, while proper nutrition supports exercise performance and recovery [8,35,36]. Bioactive compounds can enhance exercise adaptations and provide additional support for testosterone production pathways not directly addressed by exercise and diet alone [46,47].

The temporal aspects of intervention integration require careful consideration to optimize synergistic effects [8,31]. Preexercise nutrition can influence acute hormonal responses; postexercise recovery nutrition can affect adaptation processes; and the timing of bioactive compound supplementation may affect its effectiveness in supporting testosterone production [8,31,45].

Understanding individual variability in responses to combined interventions is crucial for successful clinical implementation [3,6,9]. Factors including baseline testosterone levels, genetic polymorphisms, overall health status, and lifestyle factors all influence the effectiveness of synergistic approaches, necessitating personalized protocols for optimal outcomes [6,9,54].

Practical Implementation Strategies

Clinical implementation of integrative natural approaches requires systematic assessment of individual needs, appropriate sequencing of interventions, and ongoing monitoring of responses [2,54,55]. The initial assessment should include a comprehensive evaluation of testosterone levels, overall health status, current lifestyle factors, and individual preferences that may influence adherence to the intervention [2,33,55].

The progression of interventions should typically emphasize foundational lifestyle modifications before adding supplemental bioactive compounds [2,7,56]. Exercise program development requires careful attention to individual capacity, progression rates, and integration with other life demands [8,13,30]. Nutritional interventions should focus on sustainable dietary modifications that align with individual preferences while meeting physiological requirements for testosterone optimization [34,37,45].

Monitoring protocols for integrated natural approaches should include both objective measures, such as testosterone levels, and functional assessments of strength, energy, and overall well-being [2,36,57]. Regular reassessment allows for program adjustments and optimization based on individual responses and changing needs over time [2,55].

Safety Considerations and Contraindications

Natural approaches to testosterone optimization generally demonstrate favorable safety profiles compared with pharmaceutical interventions, although certain considerations remain important for safe implementation [2,8,46]. Exercise interventions require appropriate progression to avoid injury and should be modified based on individual health conditions and physical limitations [8,26,36].

Nutritional interventions may interact with medications or existing health conditions, necessitating consultation with a healthcare provider when significant dietary modifications are implemented [33,35,51]. Bioactive compound supplementation requires attention to potential interactions, appropriate dosing, and quality control of products used [51].

The absence of significant contraindications for most natural approaches makes them suitable for broader implementation compared to testosterone replacement therapy, though individual assessment remains important for safe and effective intervention delivery [2,54]. Understanding the vicious cycle between a sedentary lifestyle and low testosterone helps guide intervention priorities and sequencing [7,32,56].

Future Directions and Research Priorities

Future research priorities should focus on clarifying the specific mechanisms through which natural interventions influence testosterone production and identifying biomarkers that predict individual responses to different intervention strategies [1,57]. Advanced research techniques, including metabolomics, proteomics, and genetic analysis, offer opportunities to better understand individual variability and optimize personalized approaches [16].

The development of standardized protocols for assessing intervention effectiveness and comparing different approaches requires continued research attention [8,29]. Establishing consensus on appropriate outcome measures, intervention durations, and response criteria will facilitate more effective clinical implementation and research translation [8,29,55].

Investigation of dose-response relationships for various natural interventions remains a critical research priority [8,29,47]. Understanding optimal intensities, frequencies, and durations for exercise interventions, appropriate dosing for nutritional strategies, and effective concentrations of bioactive compounds will enhance the precision of clinical recommendations [8,29,46].

Clinical Translation

The translation of research findings into practical clinical applications requires continued development of evidence-based protocols that can be effectively implemented in diverse healthcare settings [2,54]. Training healthcare providers in assessment techniques, intervention design, and monitoring strategies represents an important component of successful clinical translation [2,54].

Development of decision-making frameworks that guide intervention selection based on individual characteristics and preferences will enhance the clinical utility of natural approaches for testosterone optimization [54,55]. These frameworks should incorporate both evidence-based recommendations and practical considerations that influence implementation success [2,50].

Integration of natural approaches with conventional medical care requires ongoing attention to ensure appropriate patient selection, monitoring protocols, and coordination among healthcare providers involved in comprehensive care delivery [2,50]. Research examining the interaction between social support and hormonal status provides important insights for comprehensive intervention approaches [54].

Understanding the relationships among metabolic health, body composition, and testosterone levels provides additional targets for optimizing interventions [32,36,54]. Research examining the connections between sarcopenia, testosterone, and aging provides important insights for developing comprehensive intervention strategies [12,32].

Research examining the effects of multicomponent interventions that combine exercise, nutrition, and targeted supplementation provides evidence for comprehensive approaches to healthy aging [58]. Understanding the optimal integration of these modalities will inform future intervention development [8,58].

## Conclusions

Age-related testosterone decline is a significant health concern that affects metabolism, cardiovascular health, bone density, muscle mass, and psychological well-being in the aging male population. While testosterone replacement therapy offers direct hormonal supplementation, safety considerations have driven growing interest in natural approaches that preserve endogenous production capacity. The evidence supporting integrative strategies, combining exercise interventions, nutritional optimization, and bioactive compounds, demonstrates considerable promise for addressing testosterone decline through multiple complementary mechanisms. Combined resistance-aerobic training protocols provide particularly robust evidence for beneficial effects on testosterone levels while simultaneously addressing broader aspects of healthy aging. Nutritional strategies emphasizing adequate macronutrient intake, anti-inflammatory dietary patterns, and micronutrient optimization create metabolic environments conducive to endogenous testosterone production, while bioactive compounds offer targeted support for steroidogenic pathways and protection against age-related decline.

This comprehensive evidence base demonstrates that natural approaches can effectively address the complex interplay between lifestyle factors and hormonal health, offering promising alternatives to pharmaceutical interventions with favorable safety profiles and sustainable benefits for healthy aging. However, understanding individual variability in responses and developing personalized intervention protocols remains crucial for maximizing effectiveness and ensuring long-term hormonal optimization throughout extended lifespans. These integrative frameworks represent a paradigm shift toward proactive, multifaceted strategies that support both hormonal health and overall physiological resilience in aging men.
